# Molecular Basis for Global Incidence of Pemphigoid Diseases and Differences in Phenotypes

**DOI:** 10.3389/fimmu.2022.807173

**Published:** 2022-01-21

**Authors:** A. Razzaque Ahmed, Sarah Anwar, Pedro A. Reche

**Affiliations:** ^1^ Department of Dermatology, Center for Blistering Diseases, Tufts University School of Medicine, Boston, MA, United States; ^2^ Tufts University School of Medicine, Boston, MA, United States; ^3^ Department of Immunology & O2, Faculty of Medicine, University Complutense of Madrid, Madrid, Spain

**Keywords:** MHC class II genes, DQB1*;03:01, pemphigoid diseases, global incidence, phenotypic presentation, DPP4-inhibitor associated pemphigoid

## Abstract

Pemphigoid (Pg) diseases are a group of potentially fatal autoimmune mucocutaneous diseases. They have different clinical phenotypes, involving only the skin or multiple mucous membranes. They occur globally and frequently affect the elderly. The common marker among all variants is the presence of autoantibodies targeting the dermal-epidermal or mucosal-submucosal junctions, or basement membrane zone (BMZ). Four target antigens in the BMZ were studied. These included BPAG1, BPAG2 and subunits of α6 and β4 human integrins. Our objective was to find a molecular basis for the global incidence of Pg diseases and a mechanism that will explain the vast differences in clinical phenotypes and outcomes. All the variants of Pg that were analyzed had a statistically significant association with HLA-DQβ1*03:01 in ten countries on four continents. This explains the reason for global incidence. Prediction models discovered multiple peptides in each of the four antigens that serve as T cell epitopes. These T cell epitopes were shown to bind to HLA-DQβ1*03:01. In addition, structure modelling demonstrated the peptide-HLA complex bound to the T cell receptor. These autoreactive T cells would stimulate B cells to produce specific anti-BMZ autoantibodies. Anti-BMZ autoantibodies with different specificities will produce different phenotypes, which will account for involvement of different tissues and organs in different molecules. The contribution this study makes is that it provides a molecular basis of why a similar disease occurs in different racial groups. Furthermore, it provides the basis for the production of autoantibodies with different specificities, which resultantly produces different phenotypes.

## Introduction

Pemphigoid (Pg) is a group of autoimmune blistering diseases that can affect the skin and multiple mucous membranes (1). They can be fatal, difficult to diagnose, and treatment can be delayed. Data presented in this manuscript suggests that they are a good model to study autoimmunity.

The lesions in bullous pemphigoid (BP) are vesicles and bullae of variable sizes, in an acral or generalized distribution (2). In some patients mucous membranes may be involved. The most common cause of death in these patients is opportunistic infection due to prolonged iatrogenic immune suppression (3). BP affects predominantly the elderly, which is of particular relevance as the aging population is increasing globally (4). The mortality rate increases with each increasing decade of life (5). BP occurs between 2.4 to 23 cases per million in the general population worldwide (5).

Mucous membrane pemphigoid (MMP) predominantly affects multiple mucosae and not infrequently the skin. As the blisters heal, they cause scarring, which is the reason for its former name, cicatricial pemphigoid. In most patients, this scarring produces sequelae that are catastrophic and severely impact the quality of life (1, 6). Of special note are two subsets of MMP, ocular MMP and oral MMP. Other clinical entities such as anti-laminin-332 pemphigoid (7) and anti-p200 pemphigoid (8), are not included in this study. There is a general absence of data on these two disease entities, which is central to the hypothesis of this study.

The most common feature among pemphigoid diseases is that the blisters appear at the junction of the epidermis and dermis or mucosa and submucosa. Using direct immunofluorescence, the immunopathologic features include deposition of IgG and complement at the dermal-epidermal junction or basement membrane zone (BMZ) in the perilesional tissues (1). This is the standard test for diagnosis. Autoantibodies to BMZ proteins can be detected by indirect immunofluorescence using human skin or monkey esophagus as substrate (9). In some cases, ELISAs have been developed (1). In several studies anti-BMZ autoantibodies have been purified from plasma of patients with active pemphigoid diseases. When these purified human anti-BMZ autoantibodies are cultured with human tissue *in vitro*, they bind to the BMZ, when studied by histology and immunopathology. When injected into laboratory animals, they produce blisters *in vivo* (1, 10). Such studies confirm their role in the pathogenesis. In addition, multiple cells of the immune and inflammatory pathways, play a pivotal and essential role in the phenotypic presentation of pemphigoid diseases.

The pathognomonic and unique feature of MMP is that it causes scarring as it heals, except in the oral cavity (6). Scarring does not occur in BP. In the conjunctiva, the scarring leads to blindness in approximately 25% of patients (11). In the larynx, abrupt closure can result in sudden asphyxiation (6). Esophageal strictures can cause esophageal rupture and can result in fatal mediastinitis (12). Vaginal involvement can result in severe vaginal stenosis (12). Anal involvement can result in constant fecal leakage and the need for adult diapers for most of the patient’s life (12). MMP is more rare with an incidence of 1-2 cases per million (13).

In BP, the anti-BMZ autoantibodies are directed against BP antigen 1 (BPAG1 or BP230) and BP antigen 2 (BPAG2 or BP180) (14, 15). In a large cohort of patients from Germany, in a recent study, the most frequently targeted antigen was BP180 (15). Their injection produces blisters in the skin of neonatal mice (16, 17). In MMP, the antigens are a subunit of human integrins β4 and α6. Antibodies from patients’ sera against β4 produced subepidermal blisters in neonatal mice (18). When normal human oral mucosa was incubated with antibodies to subunit of human α6 integrin from sera of patients with oral pemphigoid, separation of the mucosa from the submucosa has been observed (19).

Earlier studies from our group demonstrated that patients with BP, MMP, and its subsets had a strong correlation with the HLA-DQβ1*03:01 allele, in spite of strikingly different clinical presentations, courses and prognoses (20–22).

Recently, a new group of drugs has been added to the treatment of type II diabetes mellitus, known as dipeptidyl peptidase-4 inhibitors (DPP4-is). Many patients treated with these drugs have been reported to develop pemphigoid (23).

The purpose of this study was to determine the global presence of pemphigoid diseases. Furthermore, to investigate the molecular basis for the global presence and simultaneously to study what mechanisms might explain such striking differences in clinical profiles and clinical outcomes, and the reasons for production of autoantibodies to diverse proteins in the BMZ.

## Materials and Methods

PubMed, Embase, and Medline searches were conducted using the following key words: bullous pemphigoid, mucous membrane pemphigoid, cicatricial pemphigoid, oral pemphigoid, BMZ proteins, anti-BMZ antibodies, and HLA genotyping.

### Patient Selection

Inclusion criteria included (i) the presence of the location of the study, (ii) the ethnicity or race of the patients and the control group being identical, (iii) data on HLA genotyping, (iv) clinical profile of patients to confirm the clinical diagnosis, and confirmation of diagnosis by histology and immunopathology.

Exclusion criteria included (i) inadequate or incomplete clinical description of the patients or the control group and/or absence of histology and immunopathology and (ii) presence of inappropriate (age and sex matched) control group.

### HLA Class II Genes

The data available on HLA typing results in patients with BP, MMP, and patients with diabetes mellitus treated with DPP4-i drugs who developed pemphigoid, formed the database for this study. In each study, presence of data on adequate controls was carefully analyzed.

HLA studies were conducted at different centers worldwide. The results were used in the database as provided by the authors. In two studies regarding MMP, data was reported in the studies as allele frequencies and was converted into patient frequencies (22, 24). In one study, information regarding controls was absent in the publication (20). This information was provided *via* written communication by the author.

Statistical significance of the difference in frequency of HLA DQβ1*03:01 between patients and controls was estimated by Chi-square test and Yates’ correction (SPSS 27). A p value of less than 0.05 was considered significant.

### Identification of T Cell Epitopes in Pemphigoid Antigens

Autoreactive T cells recognize self-peptide antigens bound to HLA class II (HLA II) molecules. Hence T cell epitopes may be deduced by predicting peptide binding to HLA II molecules (25). In this study, peptide binding to HLA II molecules was assessed at the RANKPEP server (http://imed.med.ucm.es/tools/rankpep.html). T cell epitopes within BP180, BP230, human β4 integrin, and α6 integrin that are restricted by HLA-DQ7 were predicted. The β chain of HLA-DQ7 is DQβ1*03:01. For the prediction of the peptide-HLA binding, RANKPEP relies on Position Specific Scoring Matrices (PSSM), which are derived from peptides that are known to bind to HLA molecules. Only those peptides that had a score higher than the Binding Threshold were considered as potential candidates for binding to DQβ1*03:01., as described earlier (26–28).

### Generation of Tertiary Structures of Potential T Cell Epitopes Bound to DQ7 (DQβ1*03:01)

The tertiary structure of DQ7 (DQA1*03:01/DQβ1*03:01) in complex with 15-mer peptide antigens FNWLPPGKPMGYRV, DNVIRKYGDPGSLFG, PAKAIAAVKSGGAVL, and LERIRRSILPYGDSM from integrin β4 (AC: NP_000204), integrin α6 isoform a (AC: NP_001073286), dystonin-1 (BPAG1) (AC: NP_899236), and alpha 1 type XII collagen (BPAG2 or BP180) (AC: NP_000485), respectively, were generated by homology modeling after two known tertiary structures of DQ8 (PDB IDs: 1JK8 & 2NNA) using a standalone version of MODELLER (29). Tertiary structure models were subjected to MODELLER energy optimization methods, selecting the best model upon discrete optimized potential energy (DOPE) scores. Superimposition of tertiary structures and molecular graphic representations were obtained using PyMol Molecular Graphics System, Version 2.0 Schrodinger, LLC.

## Results

### HLA Class II Genes in Pg Patients

Nineteen studies published between 1990 and 2020 were included in this study.

Nine reports that included data on 904 patients with BP were included in this analysis. The studies were from the following countries: Iran (30), Brazil (31), China (32–34), Britain (35), the US (20), Germany (36), and Japan (37), each reporting their native populations (**Table 1**).

**Table 1:** HLA DQB1*03:01 allele frequencies in bullous pemphigoid.

Author	Journal & Year	No. of pts	No. of alleles *0301	Pt allele frequency (%)	No. of controls	No. of alleles *0301	Control allele frequency	Countries	Race	p value
Esmaili	IJI. 2013	50	36	36	180	85	23.6	Iran	Caucasian	0.02
Chagury	Ann Dermatol. 2018	17	13	38.2	297	84	14.1	Brazil	10 white, 6 mixed, 1 black	3.72 x 10-4
Fang	JDS. 2017	105	59	28.1	420	158	18.81	China	Asian	0.003
Sun	JID. 2018	575	326	28.5	976	390	20	China	Asian	1.27 x 10-7
Banfield	BJD. 1998	73	39	26.7	604	189	15.6	Britain	Caucasian	<0.01
Delgado	PNAS USA. 1996	21	15	35.7	109	35	16.05	US	Caucasian	0.005
Budinger	JCI. 1998	15	13	43.3	24	11	22.9	Germany	Caucasian	0.05
Okazaki	J Dermatol. 2000	23	6	13.0	525	101	9.62	Japan	Asian	NS
Gao	CED. 2002	25	15	30.72	57	30	26.25	China	Asian	NS

When the frequency of HLA DQβ1*03:01 was collectively compared between BP patients and controls, it was highly statistically significant in the BP patients (p<0.00001).

In eight reports, MHC class II genes were studied in 335 patients with MMP from the US (21, 22, 38), UK (39), France (24, 40), Germany (41), and Italy (42). When the frequency of DQβ1*03:01 was compared between the patients and the controls, the frequency in the patients was highly statistically significant (p<0.00001) (**Table 2**).

**Table 2:** HLA DQB1*03:01 patient frequencies in mucous membrane pemphigoid.

Subset	Author	Journal & Year	No. of pts	No. pts DQB1 *0301	Percent	No. of controls	No. controls *0301	Percent	Countries	Race	p value
MMP	Hubner	Eye. 2018	39	31	79.5	39	13	33.3	Germany, UK	Caucasian	0.0000 9
MMP	Setterfield	BJD. 2001	128	97	75.8	177	53	29.9	France	Caucasian	<1.0 x 10-8
MMP	Drouet	EJD. 1998	25	20	80	106	46	43.4	France	Caucasian	<0.001
OCP	Chan	JID. 1997	21	16	76.2	42	14	33	US	Caucasian	<0.005
OCP	Ahmed	PNAS USA. 1991	23 haploty pes	20 haploty pes	87.0	46 haplotyp es	23 haplotyp es	50	US	Caucasian	0.003
OCP	Yunis	PNAS USA. 1994	17	13	76.5	65	22	33.8	US	Caucasian	0.002
OP	Yunis	PNAS USA. 1994	22	15	68.2	65	22	33.8	US	Caucasian	<0.001
OP	Chan	JID. 1997	22	15	68.2	42	14	33	US	Caucasian	<0.025
MMP (mainly OP)	Carrozzo	BJD. 2001	28	27	96.4	97	47	48.5	Italy	Caucasian	0.0001
MMP (mainly OP)	Walton	J Oral Pathol. 2020	15	11	73.3	10.000	3800	38	UK	Caucasian	0.005

In examining data on DPP4i-associated BP, only two studies, one from Japan (43) and one from Finland (44), MHC class II genes were detected, 30 patients in total. When the frequency of DQβ1*03:01 was compared between patients and controls, the frequency in the patients was highly statistically significant (p<0.00001).

These data clearly demonstrate that in many countries that are ethnically different from each other, the clinical profiles are similar. The MHC class II genes are identical, demonstrating the pivotal role of DQβ1*03:01 being central to the pathogenesis of BP and MMP. Despite this remarkable similarity, patients develop two very distinct clinical profiles with different clinical outcomes. As described below, different peptides within each antigen may be involved in different racial or ethnic groups. Similarly, the presence of different portions of the peptide binding to DQβ1*03:01 may produce a different clinical profile, accounting for involvement of different tissues or organs.

### Putative T Cell Epitopes in Pg Antigens

**Figure 1:**
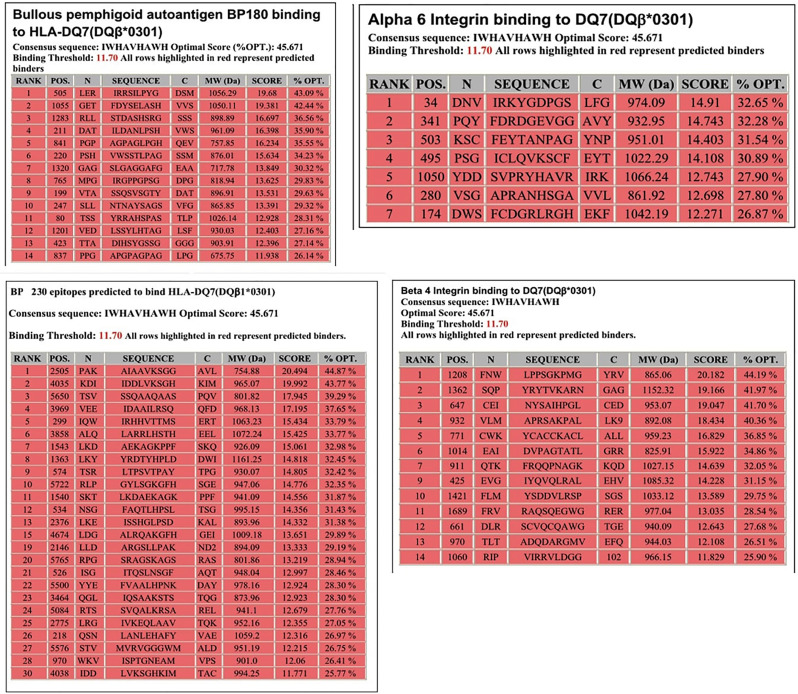
Potential HLA–DQ7 (DQβ1*03:01)-binding peptides in BPAG1, BPAG2, human β4 integrin, and human α6 integrin. Obtained from our previous publication. Autoimmunity Reviews 2011;11:40-47. With permission of the editor.

Molecular analysis of possible antigen binding sites on HLA-DQ7 (DQβ1*03:01) were done by a computer model. The computer model used identified peptides within the four antigens known to be pathogenic in pemphigoid diseases, that potentially could be T cell epitopes. Identified were 14 sites within BP180, 30 sites within BP230 14 sites within β4 integrin, and 7 sites within α6 integrin (**Figure 1**).

### T Cell Receptor Recognition of T Cell Epitopes in Pg Antigens

**Figure 2:**
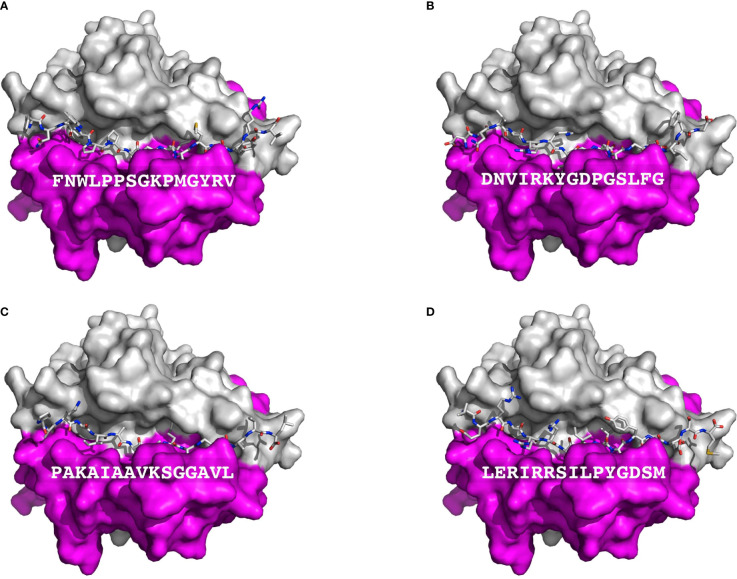
Presentation of BP autoantigens by DQ7. Figure shows a TCR view of the molecular surface of DQ7 (HLA-DQA1*03:01/HLA-DQβ1*03:01) in complex with peptide antigens, shown as sticks, from Integrin β4 **(A)**, Integrin α6 isoform a **(B)**, Dystonin-1or BPAG1 **(C)**, and alpha 1 type XII Collagen (BPAG2 or BP180) **(D)**. The amino sequence of each of peptide is shown over the molecular surface renderings. In these representations, the α and β chains of DQ7 are colored in purple and grey, respectively. Tertiary structures of peptide-DQ7 complexes were obtained by homology modeling (details in *Methods*).

**Figure 3:**
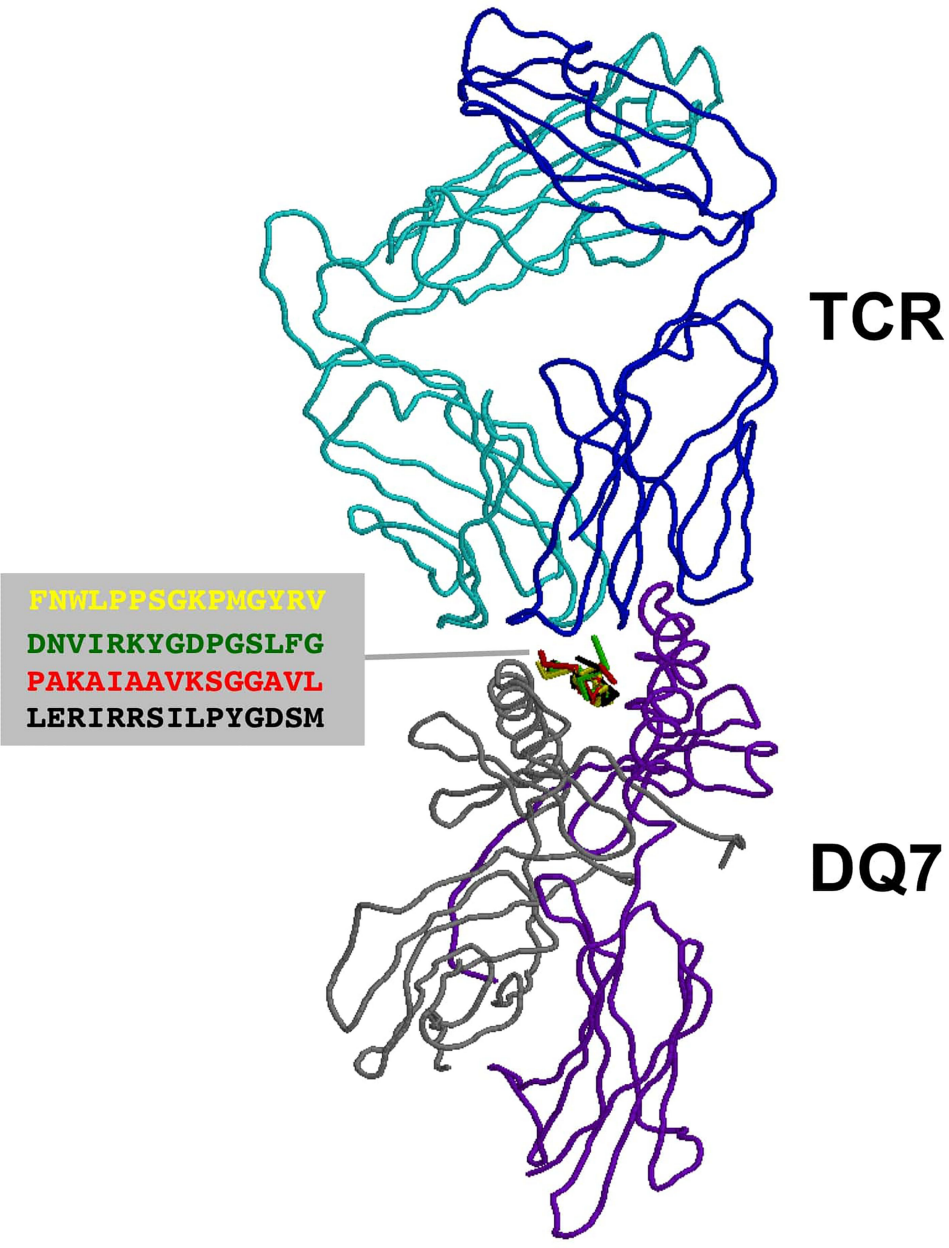
TCR recognition of autoantigens presented by DQ7. Figure shows a ribbon rendering of DQ7 with bound superimposed BP peptide antigens, being recognized by a TCR. BP peptide antigens are colored in yellow (FNWLPPGKPMGYRV), green (DNVIRKYGDPGSLFG), red (PAKAIAAVKSGGAVL) and black (LERIRRSILPYGDSM), respectively. DQ7 α and β chains are colored in purple and grey, respectively, while the α and β chains of the TCR are shown in cyan and blue, respectively. The TCR was positioned after superimposing the tertiary structure of DQ7 models with that in PDB 5KS9 corresponding to HLA-DQ8 with a bound glia-α1 peptide in complex with a TCR. Tertiary structures of peptide-DQ7 complexes were obtained by homology modeling (details in *Methods*) and superimposition of tertiary structures and molecular graphic representations were obtained using PyMol Molecular Graphics System, Version 2.0 Schrödinger, LLC.

T cell epitopes (generated by computer modeling) within pemphigoid antigens binding to DQβ1*03:01 were modeled for binding to T cell receptor. The graphic representation of this binding is presented in **Figure 2**. The molecular surface of DQ7 (DQA1*03:01/DQβ1*03:01) in complex with peptide antigen from β4 integrin (Panel A), α6 integrin (Panel B), BPAG1 (Panel C), and BPAG2 (Panel D). The amino acid sequence of one of the potential peptides that serves as an antigen, is shown over the molecular surface of DQ7 (**Figure 2**). These superimposed peptides are recognized the T cell receptor (TCR). The TCR was positioned after the superimposition of the tertiary structure of the models that in PDB 5KS9, corresponding to HLA-DQ8 with a bound glia-α1 peptide in complex with a TCR. Tertiary structures of peptide-DQ7 complexes were obtained by homology modeling (**Figure 3**). Superimposition of tertiary structures and molecular graphic representations were obtained using PyMol Molecular Graphics System, Version 2.0 Schrodinger, LLC.

## Discussion

**Figure 4:**
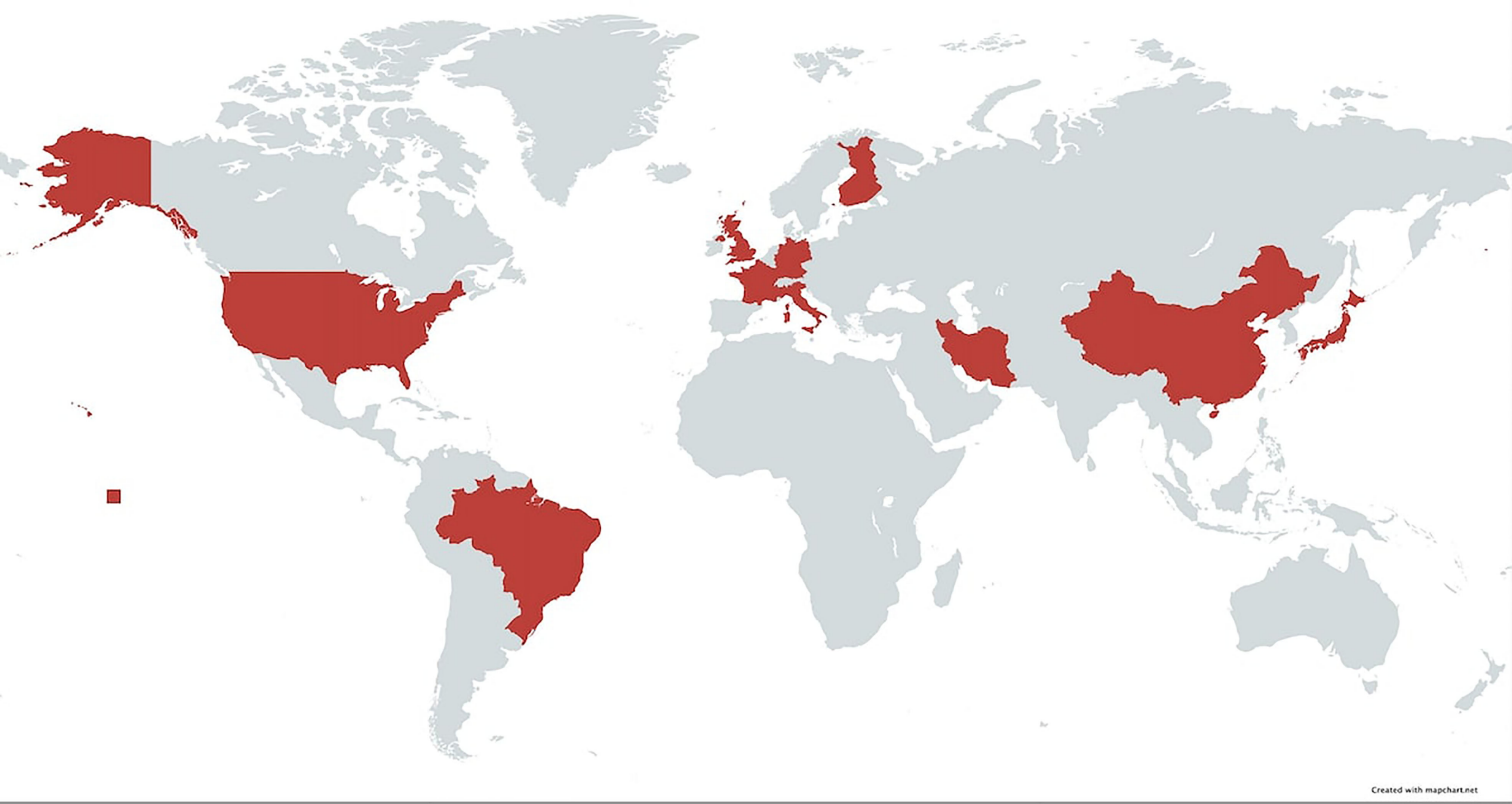
Map of the world highlighting area in which HLA studies have been done on native populations. In these countries, patients with pemphigoid diseases had a strong association with HLA-DQβ1*03:01.

In this study, we have demonstrated that the association of BP with DQβ1*03:01 has been observed in several countries spanning four continents (**Figure 4**). This confirms that the association is global and crosses racial and ethnic lines. Likewise, the association of MMP with DQβ1*03:01 is multi-national, although the studies have been done predominantly on Caucasian patients in the US, UK, and Europe. The lack of studies from Asia and Africa may in part, be due to lack of diagnostic facilities and/or availability of HLA typing laboratories. Patients with BP and MMP from both continents have been seen at the Center for Blistering Diseases. While there was no study from Australia, Pg disease must be prevalent, since 85% of the population is Caucasian of European descent (45).

One of the most recent advances in the treatment of type II diabetes mellitus is the use of DPP-4i drugs. The incidence of BP in these patients is significant (23). There are many studies that document this association, but most do not contain data on HLA typing. The two reports included in this study, from Japan and Finland, demonstrate a strong association with DPP-4i-associated BP and HLA DQβ1*03:01 (43, 44). These observations would suggest that in all likelihood, this association is also multi-national and global.

The HLA DQβ1*03:01 allele was not associated with pemphigoid diseases in two studies from Japan and China, conducted in 2000 and 2002 respectively (32, 37). However, subsequent studies from China confirmed this association to be statistically significant (33, 34). In the studies lacking this association, the absence of statistical significance could be due to the high frequency of the DQβ1*03:01 allele in the control population. In these studies, age or sex-matched controls were not used. Critical analysis of the data demonstrated that the female to male ratio was 2:1 and the majority of patients were greater than 72 years of age at the time of the onset of the diseases. Clearly, these two parameters were not present in the control population. This might in part provide an explanation for the lack of the association.

Sun et al. stratified BP patients with the HLA DQβ1*03:01 allele in the Han Chinese population into groups based on the presence of antibodies. As expected, more patients demonstrated antibodies directed against BP180 than BP230. Out of 575 patients total, 94.7% were BP180-positive and 45.6% were BP230-positive. Notably, the authors observed a significant difference in the frequency of DQβ1*03:01 between the BP180 and BP230 groups. Both groups were significantly associated with the DQβ1*03:01 allele, but this association was much stronger for the BP180-positive group (p = 5.65 x 10^-3^) (33).

Autoreactive T cell responses to BP180 have been investigated in BP patients. With regards to the DQβ1*03:01 allele, Budinger et al. observed proliferative T cell responses to BP180 were DQβ1*03:01 restricted (36).

The HLA DQβ1*03:01 allele has been associated with other conditions. Statistically significant associations between the DQβ1*03:01 allele have been observed in patients with erythema multiforme, cutaneous melanoma, gastric adenocarcinoma, and cervical cancer (46–49). BP has been associated with various neurological diseases, such as multiple sclerosis, Parkinson’s disease, dementia, and epilepsy (50). However, a statistically significant association between HLA DQβ1*03:01 has been observed only in multiple sclerosis patients in Europe, Sardinia, Spain, Korea, and Kuwait (51). None of the other neurological diseases associated with BP have a similar HLA association.

Preliminary data presented in this study demonstrates that many of those patients with diabetes mellitus treated with DPP-4i drugs who developed BP were carrying the HLA-DQβ1*03:01 allele of MHC class II genes. It is entirely possible that this provided enhanced susceptibility to develop BP. These early observations warrant further detailed studies.

Pemphigoid gestationis (HG) a rare autoimmune blistering skin disease that is associated only with pregnancy (52). HG was not included in this review as it is a separate disease entity and does not share the same HLA associations (53–55).

In this study, using a computer model, we identified several peptides in BP180, BP230, and human β4 and α6 integrins that could bind to the T cell receptor. There is data that demonstrates that such models are effective in their prediction and are similar to *in vitro* models (56). Most studies have concentrated on the NC16A domain of BP180 in the process of generating pemphigoid specific autoreactive T cells (57, 58). Researchers have discovered that peptides other than those in the NC16A domain for BP180 and other peptides in BP230 can also be involved in autoreactive T cells in pemphigoid (15, 36). Studies showed that T cells react with the entire peptide and with sequences 490-534 and 507-534 (57, 58). The computer model in this study predicted that sequence of amino acids 505-513 in BP180 and others can be putative epitopes for producing autoreactive T cells. Using a Baculovirus system, investigators identified peptides within the NC16A domain and outside this domain that functioned as T cell epitopes in the *in vitro* system. These reported peptides were also present in the computer models in this study. Likewise, studies using *in vitro* systems identified T cell epitopes in BP230 (15). Similar peptides were identified in the computer model in this study. T cell epitopes in human β4 and α6 integrins have not been identified or described. We anticipate that when such *in vitro* studies become available, they will have significant homology to peptides generated by the computer model. Collectively these studies strongly suggest that peptides generated by the computer model, as potential T cell epitopes, are similar to the *in vitro* studies and are therefore relevant and useful.

Using a computer-based model, we demonstrate a representation of how peptides from BP180, BP230, β4 integrin, and α6 integrin can be T cell epitopes presented by DQ7 (**Figure 2**). These peptides are known to have a capacity to bind to DQβ1*03:01. Further, we demonstrate by a computer model how four different peptides selected from each antigen can be presented to the T cell receptor (TCR) (**Figure 3**). The specific autoreactive CD4^+^ T cells will enable these cells to engage B cells and provide the necessary help for autoantibody production. Thus, DQ7 alone could promote the production of antibodies to four antigens in the BMZ. Consequently, the anti-BMZ autoantibodies produced will have four different specificities. These anti-BMZ autoantibodies will resultantly produce four or more different clinical phenotypes, which involve different tissue(s) or organ(s).

In our hypothesis, we modeled the tertiary structure of DQ7 in complex with selected peptides. Selected antigens were those with the largest binding scores. HLA molecules contain α and β chains. The β chain of DQ7 is HLA DQβ1*03:01, which pairs with different α chains. In the model we chose HLA DQA1*03:01 because it is the one that pairs most frequently with DQβ1*03:01 (37).

These computer models, in part, provide the molecular basis for two sets of clinical observation. First, the worldwide occurrence of an autoimmune autoantibody mediated disease with similar clinical presentation. The available data on MHC class II genes, molecular analysis of the four antigens, and the computer model used for this analysis. Second, the selective involvement of specific tissues or organs in the different subsets of pemphigoid diseases. These could in part be due to tissue or organ specific anti-BMZ autoantibodies.

The epidemiology of several autoimmune diseases have been studied and indicate their global occurrence. Some of these are rheumatoid arthritis, systemic lupus erythematosus, systemic sclerosis, myasthenia gravis, and immune thrombocytopenic purpura (ITP) (59–63). The pathogenic mechanisms have been best studied in rheumatoid arthritis and (ITP) (64, 65). In some of these diseases, such as rheumatoid arthritis, HLA associations have been described. However, there are variations in the association between different continents and ethnic groups (66).

In this study, we do not provide data to demonstrate which peptide could be used in which ethnic or racial group or which peptide is associated with which organ or tissue involvement. Nonetheless, identification of potential T cell epitopes in these four antigens associated with BP, MMP, and its clinical variants, could be potentially very beneficial for *in vitro* studies and clinical correlation. Among other advances in immunopathogenesis, these models could assist in producing disease specific, race specific, targeted therapies.

## Author Contributions

The original concept and design of the study was made by AA. The potential peptides in the four antigens that can serve as molecules presented to T cell receptors was done by PR. The latest computer analysis of binding of peptides to the T cell receptor was done by PR. SA performed a detailed literature search for all the studies on the MHC Class II genes in pemphigoid diseases. The manuscript was read and edited by all three authors.

## Funding

This study in part was supported by an unrestricted research grant from the Dysimmune Diseases Foundation.

## Conflict of Interest

The authors declare that the research was conducted in the absence of any commercial or financial relationships that could be construed as a potential conflict of interest.

## Publisher’s Note

All claims expressed in this article are solely those of the authors and do not necessarily represent those of their affiliated organizations, or those of the publisher, the editors and the reviewers. Any product that may be evaluated in this article, or claim that may be made by its manufacturer, is not guaranteed or endorsed by the publisher.
